# Blood Product Transfusion in Liver Transplantation and its Impact on Short-term Survival

**Published:** 2018-08-01

**Authors:** L. Kasraian, S. Nikeghbalian, M. H. Karimi

**Affiliations:** 1 Blood Transfusion Research Center, Higher Institute for Research and Education in Transfusion Medicine, Tehran, Iran; 2 Department of Hepatobiliary and Transplantation Surgery, Shiraz University of Medical Sciences, Shiraz Transplant Center, Shiraz, Iran

**Keywords:** Blood loss, Liver transplantation, Blood product transfusion, Survival rate

## Abstract

**Background:**

Estimation of the amount of blood products required during liver transplantation can help provision of adequate blood supply, minimize transfusion-associated complications, and plan for preventive measures in high risk patients.

**Objective:**

To investigate independent predictors of peri-operative blood product transfusion and its impact on short-term survival of liver transplant recipients.

**Methods:**

In a cross-sectional study, old charts of patients who underwent liver transplantation between March 2003 and March 2013 at Namazi Hospital, Shiraz, Iran, were reviewed. The mean amount of blood product utilized during surgery and hospital stay and the related factors, including demographic characteristics, pre-transplant laboratory data, pre-transplant clinical data, operation data, and post-transplantation data were recorded.

**Results:**

We studied 1198 patients who underwent liver transplantation. The mean±SD amounts of red blood cells, fresh frozen plasma, and platelet transfusion during surgery and hospital stay were 2.67±3.5, 2.06±3.8, and 1.6±3.8 units, respectively. The mortality rate was significantly higher in patients who received high amounts of blood products (p<0.001). The mean amount of blood products’ utilized during operation was significantly (p<0.001) decreased from 2003 to 2013.The mean amount of packed cell usage during operation and hospital stay was significantly (p<0.001) correlated with age, technique of surgery, serum albumin level, cirrhosis, blood urea nitrogen, length of operation, and prothrombin time.

**Conclusion:**

Pre-operative factors may predict blood transfusion requirements in patients undergoing liver transplantation. Therefore, evaluation of patients before operation should be considered to provide adequate blood supply and minimize transfusion-associated complications. Understanding pre-operative factors associated with rate of transfusion may help us to best utilize the limited available blood resources.

## INTRODUCTION

Liver transplantation has significantly improved the outcome of patients with acute liver failure and end-stage liver diseases (ESLD) [1, 2]. This procedure, however, requires a considerable amount of blood product transfusion because of the vascular and coagulopathic nature of the liver. Bleeding due to portal hypertension, complexity of the procedure requiring multiple anastomoses and vascular transections, predispose patients to massive hemorrhage [3]. Blood transfusion rate is considered a poor prognostic factor, which decreases patient and graft survival [4]. Poor outcome in patients may be induced by more blood loss during surgery or direct negative effects of blood transfusion such as non-infectious and infectious complications [5]. Blood transfusion increases the risk of post-operative complications, infections, longer hospital stay, respiratory complications, cytomegalovirus infection, graft failure, and patient mortality [6]. Furthermore, a previous study suggests that higher red cells (RBCs) transfusion is associated with higher level of immunosuppression, higher frequency of alloimmunization, and lower patient survival [7]. Nevertheless, it is not clear whether these complications are solely because of receiving blood products or are consequences of more complicated surgeries requiring more transfusions.

Liver transplantation has been expanded significantly during recent years highlighting the role of a good support of blood products for transfusion requirements during and after the surgery. While reservation of additional units of packed cells, platelets, plasma, and cryoprecipitate for patients who require more blood transfusion may be necessary, overestimation of the amount of blood products required may compromise blood adequacy. Therefore, a good communication between transplant team and blood bank is of paramount importance to provide enough blood products for the procedure. On the other hand, understanding pre-operative factors associated with rate of transfusion may help us to better manage the limited available blood resources [8].

Previous studies have shown a correlation between intra-operative blood loss and a number of pre-operative variables including demographic characteristics, coagulation status, and biochemical parameters [2, 3, 6]. Intra-operative blood loss has also been reported to be associated with surgical technique, surgeon’ experience, length of surgery, and hemostatic status of patient. However, other studies have failed to find any correlation [5, 7].

No transfusion guideline has so far been developed for liver transplantation. Pre-operative evaluation should include factors influencing blood requirement during the operation. Estimation of the blood products required during the procedure can help us to provide adequate blood supply, minimize transfusion-associated complications, and plan ahead of specific measures in high risk patients. Considering the importance of blood transfusion rate on patient outcome, the present study aimed to investigate the rate of blood product transfusion and its related factors in patients undergoing liver transplantation.

## PATIENTS AND METHODS

A cross-sectional study was conducted between March 2003 and March 2013 at Liver Transplantation Unit, Namazi Hospital, Shiraz, Iran. All patients who underwent liver transplantation during this period were included in the study (n=1198).

The patients’ data were collected using a well-designed data collection form, including demographic characteristics (age, sex, marital status); pre-transplant laboratory data including serum aspartate aminotransferase (AST), serum alanine aminotransferase (ALT), alkaline phosphatase, bilirubin, prothrombin time (PT), blood urea nitrogen (BUN), creatinine, hemoglobin (Hb), platelet count, hepatitis B, and hepatitis C; pre-transplant clinical data (history of previous surgery, length of disease, underlying disease, ascites, encephalopathy, and cirrhosis); operation data (length of operation); length of hospital stay; and post-transplantation one-year survival. The number of units of blood products including RBC, fresh frozen plasma (FFP), and platelet transfused during surgery and hospitalization were measured and recorded. 

The study protocol was approved by the Ethics Committee, Shiraz University of Medical Sciences. The study protocol was carried out in accordance with the Helsinki declaration as revised in Seoul 2008.

Statistical Analysis

Data were analyzed using SPSS^®^ ver 22.0 for Windows^®^ (Chicago, IL, USA). Independent-sample *Student’s t* test, and one-way ANOVA were used for statistical analysis. Poisson regression was used for evaluating the effect of studied variables (demographic characteristics, pre-transplant laboratory data, pre-transplant clinical data, operation data, and post-transplantation data) on the number of blood product utilized. A p value <0.05 was considered statistically significant.

## RESULTS

The mean±SD age of the patients was 31.1±16.8 (range: 1–75) years. Almost two-thirds (63.2%, n=757) of patients were male; 97 (8.2%) had previous history of surgery, 129 (10.9%) had encephalopathy, 416 (35.2%) had esophageal varices, and 328 (27.3%) had jaundice. The mean±SD duration of liver disease was 52.9±56 (range: 1–420) months. The mean±SD duration of operation was 390.5±85.1 minutes. More than 80% (n=966) of recipients stayed alive after one year post-operative.

The mean amounts of blood products utilized during surgery and hospital are summarized in Table 1. The mortality rate was significantly higher in recipients who received higher amounts of blood products (Table 2). 

**Table 1:** The mean±SEM amount of blood products utilized during surgery and hospital stay

Blood product (unit)	During operation	During hospital stay	Total
RBC	2.64±3.59	1.51±2.90	4.16±4.68
FFP	2.11±3.91	1.20±3.12	3.31±5.13
Platelet	1.66±3.96	0.47±2.05	2.13±1.39

RBC: Red blood cell, FFP: Fresh frozen plasma

**Table 2:** The outcome of patients according to the amount of blood and blood products transfusion. Values are mean±SEM amount of blood products utilized.

Blood product (unit)	Alive patients	Dead patients	Difference (95% CI)
Total RBC	3.74±4.23	5.92±5.91	2.18 (1.47–2.88)
Total FFP	3.06±4.56	4.36±6.99	1.30 (0.51–2.08)
Total platelet	1.74±4.23	3.85±6.87	2.11 (1.36–2.85)

RBC: Red blood cell, FFP: Fresh frozen plasma, CI: Confidence interval

Poisson regression analysis revealed a significant relationship between number of units of RBC utilized and age, albumin, presence of cirrhosis, blood urea nitrogen, length of operation, and prothrombin time (Table 3). It also showed a significant relationship between the number of units of FFP utilized and age, type of surgery, length of operation, existence of encephalopathy, existence of cirrhosis, blood urea nitrogen, albumin, and prothrombin time (Table 3). There was also a significant relationship between number of platelet units utilized and age, length of operation, pre-transplantation serum albumin, pre-transplantation BUN, and prothrombin time (Table 3).

**Table 3:** Estimation parameter of factors associated with blood products utilized during operation and hospital stay determined by Poisson regression

Blood product (dependent variable)	Independent variables	Parameter estimation	Standard deviation	p value
Total Red blood cell	Age	0.47	0.009	<0.001
	Cirrhosis	2.29	0.79	0.004
	Duration of surgery	0.02	0.001	<0.001
	PT	0.05	0.016	<0.001
	Albumin	0.34	0.09	<0.001
	BUN	0.06	0.011	<0.001
Total Fresh frozen plasma	Encephalopathy	1.04	0.50	0.037
	Cirrhosis	2.74	0.96	0.004
	Duration of surgery	0.011	0.002	<0.001
	ALT	0.001	<0.001	0.009
	PT	0.072	0.02	<0.001
	Albumin	0.24	0.10	0.024
	BUN	0.048	0.013	<0.001
Total platelet	Duration of surgery	0.012	0.002	<0.001
	PT	0.039	0.21	0.059
	Albumin	0.21	0.11	0.054
	BUN	0.05	0.014	<0.001

The mean amount of blood products utilized was not correlated with the underlying diseases.

**Figure 1:**
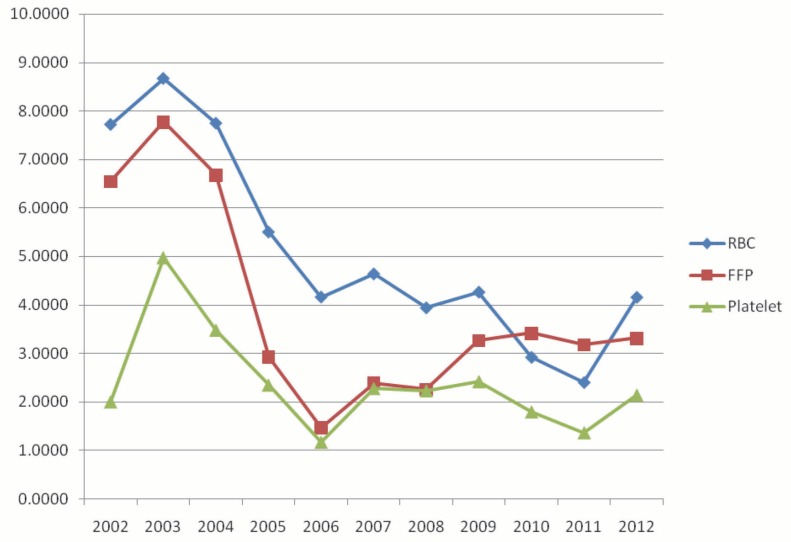
Mean amount of blood products utilized from 2002–2011.

The mean amount of blood products utilized during operation significantly (p<0.001) decreased from 2003 to 2013 (Fig 1). 

## DISCUSSION

We found that total RBCs units transfused was associated with age, pre-transplantation serum albumin level, presence of liver cirrhosis, serum BUN, length of operation, and PT. In this study, mortality was higher in those receiving higher amounts of blood products. There was also a decreasing trend in blood products utilized between 2003 and 2013.

Liver transplantation has improved outcome of patients with ESLD and acute liver failure. Although the outcome of liver recipients has improved a lot over recent years, complications that impose significant morbidity and mortality in post-transplantation period are still prevalent [9-12]. Patients with cirrhosis may develop several complications before transplantation [13]. Therefore, a through pre-transplantation evaluation of patient should be performed to early diagnose and treat these complications [14]. 

Blood transfusion is one of the factors influencing graft and patient survival. Poor outcome of patients who received more blood transfusion might be attributed to more blood loss during transplantation, or adverse transfusion reaction, infectious contamination of blood products, or immune modulation of the transfused patient [15]. One-year survival rate is decreased in patients who received any amounts of FFP or more than four units of RBC [16]. However, blood transfusions are not the only variables that can influence the survival rate.

Identifying the factors associated with transfusion requirements can help us planning for sufficient blood. The lower blood products utilized might be due to using different anesthetic and surgical methods and better blood salvage techniques [17]. In our study, blood utilization has significantly decreased from 6.09 to 2.67 units. Similar finding was reported from previous studies [18]. This reduction in blood utilization may be attributed to advances in surgical and anesthetic methods, improved graft preservation and organ allocation, better patient monitoring using the piggyback technique for surgery, and better intra-operative blood salvage [19].

Previous studies found that the amount of blood usage may be associated with the severity of the disease, etiology of liver disease, pre-operative PT, history of abdominal operation, duration of surgery, factor V levels, presence of ascites, elevated serum creatinine, elevated BUN, age, MELD score, initial fibrinogen, initial central venous pressure, total anesthesia time, presence of other hematological abnormalities such as anemia and thrombocytopenia, and history of upper abdominal surgery [2, 10, 12-18]. Furthermore, portal vein hypoplasia, decreased donor liver size, use of a graft with a raw surface, poor graft-recipient body weight ratio, poor graft preservation, prolonged time of cold ischemia, and poor quality cadaveric graft might develop non-functional liver after transplantation requiring more complex surgeries needing more amounts of transfusion [16, 19].

Whether presence and degrees of coagulopathy are related to transfusion requirement is controversial. We showed that pre-operative PT was correlated with higher transfusion rate. Similar findings were reported in another study [20]. However, other studies found no association between pre-operative coagulation indices and blood transfusion requirement [21, 22]. 

We found that the mean RBC units used was correlated with BUN. Previous studies also showed renal function as a predictive factor for estimating blood requirement [23, 24]. Renal dysfunction may cause anemia, platelet dysfunction, defective hemostasis, and bleeding, which may cause more blood utilization during liver transplantation [25].

Previous studies indicate a positive correlation between MELD score and blood products consumption [26]. High MELD score is an indicator of multisystem dysfunction and coagulopathy [26]. In general, patients with more advanced liver disease are more prone to blood loss because of portal hypertension and defective hemostasis. However, another study shows that transfusion requirement is not related to MELD score, history of previous upper abdominal surgery, pre-operative coagulation defects, and hemoglobin level [27].

Our study revealed no correlation between hemoglobin level and blood utilization. Similar result was found in another study [21]. However, in other previous studies, a negative correlation was observed between pre-operative hemoglobin level and blood requirement [28, 29]. Another study shows that correction of hemoglobin level before operation might reduce the transfusion need during transplantation [29].

We found that FFP utilization was higher in patients with encephalopathy, cirrhosis, longer duration of surgery, elevated AST, increased PT, and lower serum albumin level. A previous study demonstrates that INR and PT, which represent patients’ hemostatic status, were positively correlated with FFP consumption, which was in agreement with the findings of the current study [30]. Another study shows that FFP consumption is negatively correlated with hemoglobin level, but this correlation was not confirmed in our study [30]. Another study shows that post-transplantation survival of patients is related to the number of FFP units transfused to patients [31]. Besides, the authors found that survival rate is more correlated with FFP transfusion compared with RBC transfusion [31]. Therefore, FFP must be transfused cautiously.

In conclusion, it seems that blood transfusion requirement in patients undergoing liver transplantation was correlated with several pre-operative factors. Therefore, decision for blood requirement must be individualized for each patient by evaluating pre-operative factors to provide adequate blood supply and minimize transfusion-associated complications.
